# The association of carotid artery atherosclerosis with the estimated excretion levels of urinary sodium and potassium and their ratio in Chinese adults

**DOI:** 10.1186/s12937-021-00710-8

**Published:** 2021-06-06

**Authors:** Shuang Peng, Jiangang Wang, Yuanming Xiao, Lu Yin, Yaguang Peng, Lin Yang, Pingting Yang, Yaqin Wang, Xia Cao, Xiaohui Li, Ying Li

**Affiliations:** 1 Department of Health Management, The Third Xiangya Hospital, Central South University, Changsha, Hunan China; 2 State Key Laboratory of Cardiovascular Disease, Fuwai Hospital, National Center for Cardiovascular Diseases, Peking Union Medical College & Chinese Academy of Medical Sciences, Beijing, China; 3 Center for Clinical Epidemiology and Evidence-based Medicine, Beijing Children’s Hospital, Capital Medical University, National Center for Children Health, Beijing, China; 4 Department of Cancer Epidemiology and Prevention Research, Cancer Care Alberta, Alberta Health Services, Calgary, AB Canada; 5 Departments of Oncology and Community Health Sciences, Cumming School of Medicine, University of Calgary, Calgary, AB Canada; 6 Hunan Key Laboratory for Bioanalysis of Complex Matrix Samples, Changsha, Hunan China; 7 Department of Pharmacology, Xiangya School of Pharmaceutical Science, Central South University, Changsha, Hunan China

**Keywords:** Carotid plaque, Intima-media thickness (IMT), Estimated urinary sodium excretion (eUNaE), Estimated urinary potassium excretion (eUKE)

## Abstract

**Background:**

Arterial stiffness is an independent cardiovascular risk factor. However, the association between sodium/potassium intake and vascular stiffness was inconsistent. Therefore, a large community-based cross-sectional study was performed to try and achieve more definitive conclusion.

**Methods:**

Urinary sodium, potassium, and creatinine levels were tested in spot urine samples during physical examinations of each recruited participant. The 24-h estimated urinary sodium excretion (eUNaE) and estimated urinary potassium excretion (eUKE) levels were determined using the Kawasaki formula (used as a surrogate for intake). Carotid intima-media thickness (IMT) and plaques were measured using ultrasound.

**Results:**

In 13,523 subjects aged 18–80 years, the relationships between carotid plaques and IMT with eUNaE, eUKE and their ratios were analyzed. Overall, 30.2% of participants were diagnosed with carotid artery plaques. The ratio of estimated sodium vs. potassium excretion (Na/K ratio) of the individuals with carotid artery plaques was significantly higher than that of participants without plaque (2.14 ± 0.73 vs. 2.09 ± 0.61, *P* < 0.01). After adjusting for age, gender, and other lifestyle covariates, a significant positive relation was found between carotid plaque and Na/K ratios (OR = 1.06, *P* < 0.05). In participants without plaque, a similar positive association was observed between Na/K ratios and increased bifurcation carotid IMT (β = 0.008, *P* < 0.01), especially in the females (P_interaction_ < 0.01).

**Conclusions:**

In this study, in which sodium intake was estimated on the basis of measured urinary excretion, high estimated excretion levels of urinary sodium and/or low estimated excretion levels of urinary potassium might be associated with an increased presence of carotid atherosclerosis in Chinese individuals.

**Supplementary Information:**

The online version contains supplementary material available at 10.1186/s12937-021-00710-8.

## Introduction

Low sodium and high potassium intake is recommended as a primary prevention and control strategy to reduce the risk of hypertension and subsequent cardiovascular diseases [1–6]. However, some studies failed to identify an association between dietary sodium intake and cardiovascular events [7–9]. It appears that subsets of the population, namely African-American, elderly, obese, and hypertensive individuals, have increased sodium sensitivity [10]. Another reason is that different levels of sodium and potassium intake may have different results. In the PURE study, an estimated sodium intake of 3–6 g per day was associated with a lower risk of death and cardiovascular events than either a higher or lower estimated level of intake [6]. However, most previous studies were performed in Western countries, and there is insufficient evidence in China, a country with a high sodium and low potassium dietary pattern [11–16]. Therefore, it is important to determine whether the intake of sodium and potassium is associated with the risk of cardiovascular disease (CVD) in the Chinese population.

There are two main approaches to estimate sodium intake, namely, measuring urinary excretion or assessing dietary intake [17]. Twenty-four hour urinary sodium excretion is the reference standard for sodium and potassium intake estimation on the premise that the vast majority of sodium and potassium ingested is excreted in the urine. Dietary assessment may be measured using 24 h dietary recall, food diaries, or food frequency questionnaires. However, limitations include recall bias, variations in sodium content of common food items, lack of information on sodium added at the table or during cooking, and imprecision with estimating portion size [18, 19]. Thus, in most studies, sodium and potassium intake were estimated on the basis of measured urinary excretion [5, 6, 11–16, 20].

Atherosclerosis is an early stage of cardiovascular disease. Carotid artery intima-media thickness (IMT) is an accepted subclinical atherosclerotic marker and a predictor of future cardiovascular disease [21]. Furthermore, carotid plaque is believed to be a more stronger predictor of chronic heart disease (CHD) than carotid IMT [22, 23]. Njoroge JN et al. investigated the association of carotid IMT with 24-h urinary sodium in normotensive overweight and obese adults [24]. One study [25] indicated a positive association of spot urine levels of sodium and the ratio of estimated urinary sodium vs. potassium excretion (Na/K ratio) with carotid IMT and plaque, but that relationship was not significant after adjusting for potential confounding factors in a small sample of middle-age and elderly Chinese adults. Whether this effect can be validated in a large sample that includes young and middle-aged people needs further study. Therefore, an institution-based cross-sectional study was performed to evaluate the associations of carotid IMT and plaque with estimated urinary sodium excretion (eUNaE), estimated urinary potassium excretion (eUKE), and the Na/K ratio in physical examination samples at the Third Xiangya Hospital located in Changsha, China.

## Methods

### Study design and participants

This study was a physical examination- and institution-based cross-sectional study. The current study was performed from August 2017 to November 2018 in the Department of Health Management, the Third Xiangya Hospital. Six internal medicine physicians (including 2 resident physicians and 4 attending physicians) performed physical examinations including weight, height, systolic blood pressure (SBP), and diastolic blood pressure (DBP). Six experienced ultrasonography technicians (including 1 resident physician and 5 attending physicians) performed all of the ultrasound examinations. Blood samples were collected to measure fasting serum glucose (FSG), total cholesterol (TC), triglyceride (TG), low-density lipoprotein cholesterol (LDL-C), and high-density lipoprotein cholesterol (HDL-C) levels. Random urine samples were collected on the day of physical examination to test sodium, potassium, and creatinine excretions. Additionally, the collected information included personal details, health-related habits, family history, and self-reported disease history (such as hypertension, diabetes, stroke, and coronary artery diseases), which were obtained from the National Unified Physical Examination Questionnaire [16].

### Quality assurance of the clinical and laboratory data

Participants with SBP/DBP ≥ 140/90 mmHg or a self-reported history of hypertension or blood pressure medication use were defined as hypertensive individuals. Following the Chinese Guidelines for the Prevention and Treatment of Hypertension, seated blood pressure was measured using a mercury sphygmomanometer or electronic sphygmomanometer at least two times.

The carotid artery test was performed using the Siemens Acuson SequoiaTM 512 Ultrasound System (Mountain View, CA, USA) with a 12-MHz (9–14) linear matrix array transducer while the patient was in a supine position. Carotid IMT was identified as the distance between the leading edge of the lumen-intima echo and the leading edge of the media-adventitia echo, which was measured at the common carotid artery (CCA) and bifurcation carotid artery (BIF) on the left and right sides in longitudinal views. Carotid IMT was defined as 0.4–1.5 mm [26]. Plaque was defined as carotid IMT over 1.5 mm or focal thickening that was 50% greater than the surrounding wall thickness [27].

All blood and urine samples were analyzed using 7600 and 7170 Hitachi automatic biochemical analyzers. Fasting blood samples were collected to measure FSG, TC, TG, LDL-C, and HDL-C levels using LEADMAN test kits (Beijing LEADMAN Biochemical Co., Ltd., China), and the serum creatinine (SCr) level using Wako L-Type Creatinine M kits (Wako Pure Chemical Industries, Ltd., Japan). The sodium and potassium levels were examined using an ion selective electrode method. In the current study, the Kawasaki formulas [28] were used to estimate 24-h urinary sodium and potassium excretion from spot urine samples, and these estimates were used as surrogates for intake [6, 12, 13, 15, 20]. An international validation study reported an intraclass correlation of Kawasaki formula to actual 24 h urine collections of 0.71 [29]. Therefore, the estimates formula was used as surrogates for sodium and potassium intake in the current study.

Dyslipidemia was defined as meeting any one of the following criteria: 1) TC ≥6.22 mmol/L, 2) LDL-C ≥ 4.14 mmol/L, 3) HDL-C < 1.04 mmol/L, 4) TG ≥2.26 mmol/L, or 5) having a history of dyslipidemia or taking lipid-lowering medications.

Diabetes was defined as meeting any one of the following criteria: 1) FSG ≥7.0 mmol/L or 2) having a history of diabetes or taking antidiabetic medications.

### Ethics statement

All subjects gave their written informed consent for inclusion before they participated in the study. The available data without sensitive personal information, such as name or personal identification was acquired from the physical examination system. This study complied with the Declaration of Helsinki. The Institutional Review Board (IRB) of the Third Xiangya Hospital, Central South University (No. 2018-S393) approved the study. The IRB members included community representatives and medicine and law experts.

### Statistical analyses

All statistical analyses were performed using SAS 9.4 (SAS Institute Inc., Cary, North Carolina, USA). Continuous variables are shown as the means ± standard deviation (SD), and categorical variables are shown as percentages (%) and numbers (*n*). Differences between those with and without carotid plaques were compared using the to-sample non-parameter Wilcoxon test and the Chi-square tests for categorical variables. Linear trend across increasing Na/K ratio was tested by assuming the values of Na/K ratio as continuous variables. Odds ratios (ORs) and their corresponding 95% confidence intervals (CIs) were calculated to assess the associations of the carotid plaque risk with the Na/K ratio in logistic regression models, and linear regression models were used to compute the changes in CCA-IMT and BIF-IMT and the corresponding 95% CIs per unit increase in the ratio of estimated urinary sodium to potassium excretion. We constructed the following models sequentially: (1) not adjusted for other factors (Model 1); (2) further adjusted for lifestyle risk factors: age, sex, body mass index, smoking, and alcohol consumption (Model 2); and (3) further adjusted for hypertension, diabetes mellitus, dyslipidemia and cardiovascular disease (Model 3). In assessing associations of the Na/K ratio with carotid plaques and IMT, the influence of sex (female or male), age (≤45 years, >45 years), body mass index (BMI) (< 25 kg/m^2^ or ≥ 25 kg/m^2^), smoking status (yes or no), alcohol intake status (yes or no), hypertension status (yes or no), dyslipidemia (yes or no) and diabetes mellitus (yes or no) was investigated using tests of interaction adjusted for the above-mentioned covariates. All *P* values were 2-tailed.

## Results

**Fig. 1:**
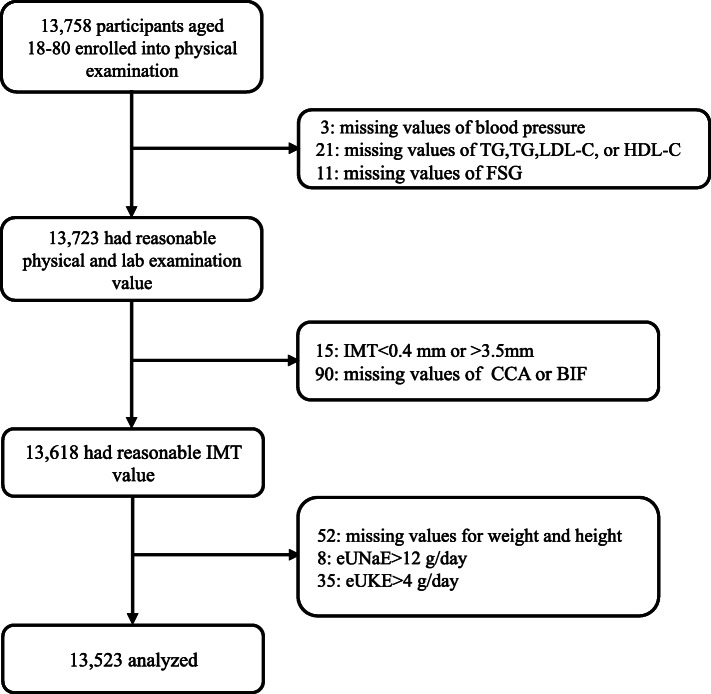
Flow chart of participant selection. Note: TC, total cholesterol; TG, triglyceride; LDL-C, low-density lipoprotein cholesterol; HDL-C, high-density lipoprotein cholesterol; FSG, fasting serum glucose; IMT, Intima-media thickness; CCA, common carotid artery; BIF, bifurcation carotid artery; eUNaE, estimated urinary sodium excretion; eUKE, estimated urinary potassium excretion

A total of 13,758 individuals attended routine health check-ups, including carotid artery ultrasound tests, between August 2017 and November 2018 at the Health Management Center and provided urine samples on the day of the physical examination for the sodium, potassium, and creatinine excretion tests; among these participants, missing or implausible values were exclude. As a result, 13,523 participants were included in the analyses (see Fig. 1).

Overall, the mean age of the 13,523 eligible participants was 48.66 ± 10.46 years old. A total of 62.6% of the participants (*n* = 8463) were male. A total of 30.2% of the participants were diagnosed with carotid artery plaque. Participants with carotid artery plaque were significantly older than those without plaque (55.31 ± 9.00 vs. 45.79 ± 9.71 years, *P < 0.001*). Individuals with carotid artery plaque were more likely to have higher BMI (24.75 ± 3.04 vs. 24.56 ± 3.25 kg/m^2^, *P = 0.002*), SBP (130.60 ± 17.49 vs. 123.04 ± 15.23 mmHg, *P < 0.001*), DBP (79.41 ± 11.72 vs. 76.01 ± 11.31 mmHg, *P < 0.001*), FSG (6.01 ± 1.89 vs. 5.56 ± 1.21 mmol/L, *P < 0.001*), TC (5.27 ± 1.00 vs. 5.05 ± 0.97 mmol/L, *P < 0.001*), TG (1.98 ± 1.64 vs. 1.90 ± 1.85 mmol/L, *P = 0.013*) and LDL-C (3.04 ± 0.88 vs. 2.85 ± 0.83 mmol/L, *P < 0.001*) than normal individuals, respectively. Moreover, those who had carotid artery plaque had lower HDL-C levels (1.33 ± 0.30 vs. 1.35 ± 0.31 mmol/L, *P =* 0.001). There was no difference in eUNaE between the two groups (4.32 ± 1.14 vs. 4.32 ± 1.15 g/day, *P =* 0.56), while individuals with carotid artery plaque were more likely to have lower eUKE levels than those without carotid artery plaque (2.08 ± 0.45 vs. 2.13 ± 0.46 g/day, *P < 0.001*). The percentages of males (68.8% vs. 59.9%, *P < 0.001*), current smokers (31.9% vs. 25.7%, *P < 0.001*) and current alcohol users (34.6% vs. 32.4%, *P = 0.006*) in the carotid artery plaque group were significantly higher than those in the group without plaque. More detailed results are presented in Table 1. Furthermore, the characteristics of participants without carotid plaque in different Na/K ratios were shown in Table S1.

**Table 1:** Characteristics of participants with or without carotid artery plaque

Characteristics (mean ± SD)	Total (***N*** = 13,523)	Without plaque (***N*** = 9444)	With plaque (***N*** = 4079)	***P***^**1**^
Age (years)	48.66 ± 10.46	45.79 ± 9.71	55.31 ± 9.00	< 0.001
BMI (kg/m^2^)	24.62 ± 3.19	24.56 ± 3.25	24.75 ± 3.04	0.002
SBP (mmHg)	125.32 ± 16.32	123.04 ± 15.23	130.60 ± 17.49	< 0.001
DBP (mmHg)	77.04 ± 11.54	76.01 ± 11.31	79.41 ± 11.72	< 0.001
FSG (mmol/L)	5.69 ± 1.46	5.56 ± 1.21	6.01 ± 1.89	< 0.001
TC (mmol/L)	5.11 ± 0.98	5.05 ± 0.97	5.27 ± 1.00	< 0.001
TG (mmol/L)	1.92 ± 1.79	1.90 ± 1.85	1.98 ± 1.64	0.013
LDL-C (mmol/L)	2.91 ± 0.85	2.85 ± 0.83	3.04 ± 0.88	< 0.001
HDL-C (mmol/L)	1.34 ± 0.31	1.35 ± 0.31	1.33 ± 0.30	0.001
Estimated UNa (g/day) ^2^	4.32 ± 1.15	4.32 ± 1.15	4.32 ± 1.14	0.56
Estimated UK (g/day) ^2^	2.11 ± 0.45	2.13 ± 0.46	2.08 ± 0.45	< 0.001
Na/K ratio	2.10 ± 0.65	2.09 ± 0.61	2.14 ± 0.73	< 0.001
Male sex (%)	37.4	40.1	31.2	< 0.001
Current alcohol users (%)	33.1	32.4	34.6	0.006
Current smokers (%)	27.6	25.7	31.9	< 0.001
Hypertension^3^	32.6	26.1	47.7	< 0.001
Dyslipidemia^4^	37.5	34.9	43.3	< 0.001
Diabetes mellitus^5^ (%)	8.3	6.0	13.4	< 0.001
CVD (%)	1.6	1.0	3.1	< 0.001

Note: *SD* standard deviation, *BMI* body mass index, *SBP* systolic blood pressure, *DBP* diastolic blood pressure, *FSG* fasting serum glucose, *TC* total cholesterol, *TG* triglyceride, *LDL-C* low-density lipoprotein cholesterol, *HDL-C* high-density lipoprotein cholesterol, *UNa* urinary sodium excretion, *UK* urinary potassium excretion;

^1^ *P* were obtained between those with or without plaque using the two-sample nonparametric Wilcoxon test and the chi-square test for categorical variables

^2^ 24-h urinary sodium, potassium, and creatinine levels were estimated using the Kawasaki formula

^3^ Hypertension was defined as self-reported hypertension diagnosed by a physician, self-reported regular use of antihypertensive medications, or systolic/diastolic blood pressure at recruitment ≥140/90 mmHg

^4^ Dyslipidemia was defined as meeting any of the following criteria: 1) TC ≥ 6.22 mmol/L; 2) LDL-C ≥ 4.14 mmol/L; 3) HDL-C < 1.04 mmol/L; 4) TG ≥2.26 mmol/L; 5) self-reported dyslipidemia or use of lipid-lowering medications;

^5^ Diabetes mellitus was defined as self-reported diabetes diagnosed by a physician, self-reported regular use of antidiabetic medications, or fasting glucose at recruitment ≥7.0 mmol/L.

**Fig. 2:**
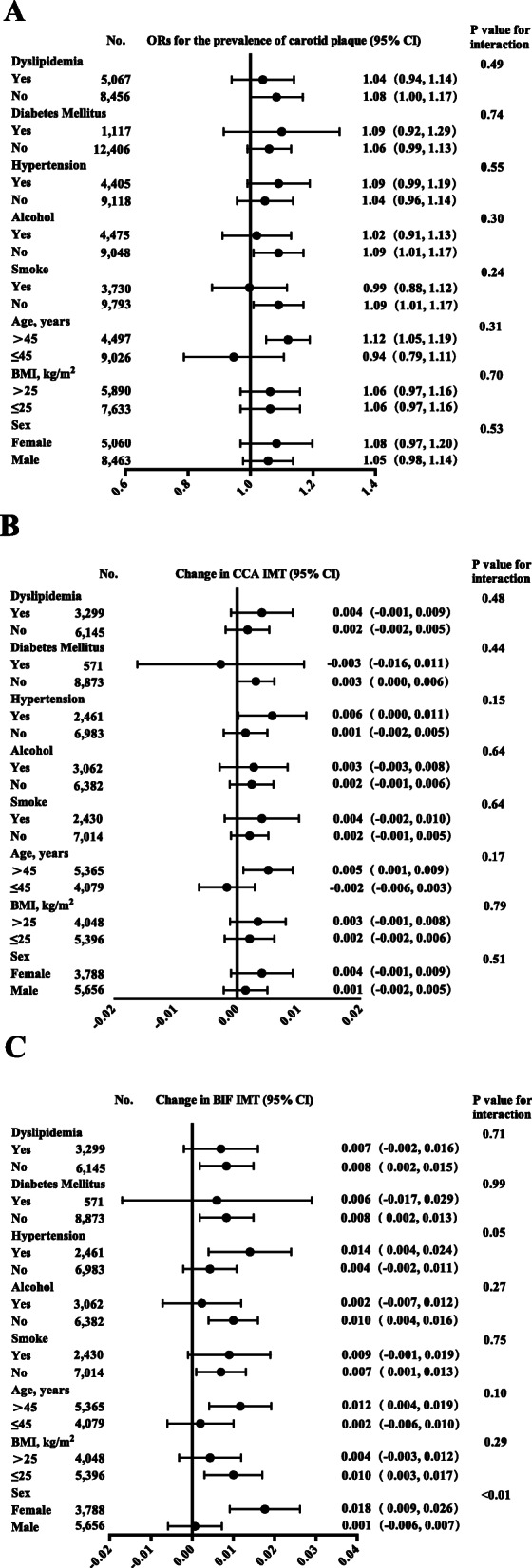
Forest plots of changes in carotid plaque and IMT per unit increase in ratio for estimated urinary sodium to potassium excretion. Note: BMI, body mass index; carotid IMT, carotid artery intima-media thickness; CCA-IMT, common carotid artery intima-media thickness; BIF-IMT, bifurcation carotid artery intima-media thickness; Na/K ratio, ratio of estimated urinary sodium excretion to potassium excretion. Adjusted for age, sex, BMI, smoking, alcohol use, hypertension, diabetes mellitus, dyslipidemia and cardiovascular disease

As shown in Table 2, a significant negative association was observed between eUKE and the incidence of carotid artery plaque in the logistic regression of Model 1(*OR* = 0.81, *P*<*0.01*), while a significant positive association was found between the Na/K ratio and the incidence of carotid artery plaque (*OR* = 1.14, *P*<*0.01*) in the logistic regression of Model 1. After adjusting for confounding factors (age, sex, BMI, smoking, and alcohol drinking in model 2, plus hypertension, diabetes mellitus, dyslipidemia and cardiovascular disease in model 3), a positive marginal association was also observed between the Na/K ratio and the incidence of carotid artery plaque in Models 2 and 3 (*OR* = 1.07, *P = 0.03* and *OR* = 1.06, *P = 0.048* in models 2 and 3, respectively). No significance relationship was shown between eUKE and carotid plaque after adjusting for confounding factors. Furthermore, no dose-response trend of carotid IMT with the Na/K ratio increase was found (See Table S2). The stratified analysis of the association of the Na/K ratio with carotid artery plaque is shown in Fig. 2A. No significant differences were found in the associations between Na/K ratio and carotid artery plaque between the subgroups by age, BMI, personal habits and chronic status (all *P*interaction > 0.05).

**Table 2:** Adjusted associations of carotid plaque and IMT with eUNaE, eUKE and the Na/K ratio

Variables	eUNaE			eUKE			Na/K ratio		
**Carotid Plaque**									
	**OR**	**95% CI**	***P***	**OR**	**95% CI**	***P***	**OR**	**95% CI**	***P***
Model 1 (*n* = 13,523)	0.99	0.96, 1.03	0.68	0.81	0.74, 0.88	< 0.01	1.14	1.08–1.21	< 0.01
Model 2 (*n* = 13,523)	1.03	0.99, 1.07	0.12	1.00	0.91, 1.10	0.99	1.07	1.01–1.14	0.03
Model 3 (*n* = 13,523)	1.03	0.99, 1.07	0.11	1.02	0.93, 1.12	0.69	1.06	1.00–1.13	0.048
**CCA-IMT**									
	**β**	**95% CI**	***P***	**β**	**95% CI**	***P***	**β**	**95% CI**	***P***
Model 1 (*n* = 9444)	0.004	0.002, 0.006	< 0.01	0.004	−0.001, 0.008	0.12	0.006	0.002, 0.009	< 0.01
Model 2 (*n* = 9444)	0.002	0.000, 0.003	0.04	0.001	−0.003, 0.005	0.62	0.003	0.000, 0.006	0.09
Model 3 (*n* = 9444)	0.002	0.000, 0.004	0.04	0.001	−0.003, 0.006	0.54	0.003	0.000, 0.005	0.10
**BIF-IMT**									
	**β**	**95% CI**	***P***	**β**	**95% CI**	***P***	**β**	**95% CI**	***P***
Model 1 (*n* = 9444)	0.004	0.000, 0.007	0.03	−0.005	−0.013, 0.003	0.23	0.013	0.007, 0.019	< 0.01
Model 2 (*n* = 9444)	0.001	−0.002, 0.004	0.61	−0.009	−0.016, − 0.001	0.02	0.008	0.003, 0.013	< 0.01
Model 3 (*n* = 9444)	0.001	−0.002, 0.004	0.60	−0.008	−0.015, − 0.001	0.03	0.008	0.003, 0.013	< 0.01

Model 1 was not adjusted for other factors

Model 2 was adjusted for age, sex, body mass index, smoking, and alcohol consumption

Model 3 was adjusted for age, sex, body mass index, smoking, alcohol consumption, hypertension, diabetes mellitus, dyslipidemia and cardiovascular disease

Multivariable linear regression demonstrated a significant positive association of CCA-IMT with eUNaE and the Na/K ratio in model 1 (β = 0.004, *P* < 0.01 and β = 0.006, *P* < 0.01, respectively). A marginal increase in CCA-IMT was observed for each increase in eUNaE (increment of 0.002 mm, 95%CI:0.000 to 0.003, increment of 0.002 mm, 95%CI:0.000 to 0.004, respectively) after adjusting for confounding factors in models 2 and 3. A negative association of BIF-IMT with eUKE was observed after adjusting for confounding factors in models 2 and 3 (increment of -0.009 mm, 95%CI:-0.016 to − 0.001, increment of -0.008, 95%CI:-0.015 to -0.001, respectively). Moreover, the positive associations of BIF-IMT with the Na/K ratio were observed in the three models (increment of 0.013 mm, 95%CI:0.007 to 0.019, increment of 0.008 mm, 95%CI:0.003 to 0.013, and increment of 0.008 mm, 95%CI:0.003 to 0.013, respectively). More detailed results are presented in Table 2. Furthermore, Fig. 2B and C illustrate the forest plots of the change in CCA-IMT and BIF-IMT per unit increases in the Na/K ratio across various subgroups. The positive association of the Na/K ratio with BIF-IMT was more significant in females (increment of 0.018 mm, 95%CI: 0.009 to 0.026) than in males (increment of 0.001 mm, 95%CI: -0.006 to 0.007, *P*interaction < 0.001). No significant differences were found in the associations between the Na/K ratio and IMT between the subgroups by age, BMI, personal habits and chronic status (all *P*interaction > 0.05).

## Discussion

The present cross-sectional study of a relatively young Chinese population confirmed the association between estimated sodium and potassium excretion (used as surrogates for intake) and carotid artery atherosclerosis independent of demographic and socioeconomic factors, smoking and alcohol drinking, and cardiometabolic factors. Therefore, sodium restriction and potassium addition might be effective in preventing carotid atherosclerosis.

Traditional risk factors for carotid atherosclerosis primarily include age, smoking, hypertension, diabetes and dyslipidemia. The benefits of lower sodium intake for cardiovascular events were strengthened recently in populations with a high sodium intake [12, 13, 20]. Our study found that increased excretion of urinary sodium was marginally associated with a greater incidence of common carotid artery wall thickening independent of demographic, socioeconomic and common disease factors. Similar results were observed in several observational studies. A cross-sectional study found that 24-h urinary sodium excretion was positively associated with carotid IMT in normotensive overweight and obese adults [24]. Another cross-sectional study of middle-aged and older Chinese populations confirmed a positive association between urinary excretion of sodium and the presence of carotid atherosclerosis [25].

The relationship between urinary potassium excretion and carotid atherosclerosis was analyzed. The results suggest that urinary potassium excretion had no relationship with carotid plaque, but BIF-IMT remarkable decreased with increasing urinary potassium excretion after correction for confounding factors. Previous studies on urinary potassium excretion and cardiovascular events led to inconsistent results. Dai’s study showed that urinary potassium excretion had no relationship with carotid IMT [25]. A meta-analysis of 11 prospective studies demonstrated that an increase in potassium intake of 1.64 g/day (42.1 mmol/day) was associated with a 21% reduction in the risk of stroke (95% CI, 10 to 32%), might also reduce the risk of CHD and total CVD [30]. Another meta-analysis confirmed the inverse association between potassium intake and stroke risk, with a potassium intake of 90 mmol/day being associated with the lowest risk of stroke [31]. Therefore, more studies are needed to address the potential effects of potassium.

Although increased urinary sodium or potassium excretion alone did not decrease the risk of carotid plaque in the current study, an increased urinary Na/K ratio was definitely associated with a greater incidence of carotid plaque and BIF wall thickening in our participants, even after the confounding factors were corrected. However, this phenomenon was not present in CCA-IMT, which may be because BIF is more prone to wall thickening and plaque formation [32]. These results suggest that the combination of a low-sodium and high-potassium diet may be associated with a healthy carotid artery. Unfortunately, the Chinese diet is predominantly high in sodium and low in potassium [11–16]. A high sodium and low potassium diet may be the initiating factor of carotid intima-media thickening, followed by the induction of oxidative stress, chronic inflammation [33–35] and other factors, further promoting carotid intima-media thickening. Therefore, the Chinese government should pay extensive attention to potassium addition strategies, beyond sodium intake restrictions, in the future.

The association of urinary sodium and the Na/K ratio with IMT was found in different gender subgroups. In this study, the association between Na/K ratio and BIF-IMT tended to be more obviously in female subjects. It is unclear whether different gender populations vary in their susceptibility to sodium and potassium intake. Further studies are needed to clarify this issue. A previous study [25] found that the association between urinary sodium/creatinine ratio and carotid IMT tended to be more significant in normal-weight individuals. But our study failed to confirm this result. Moreover, the previous studies were shown salt restriction could reduce the risk of cardiovascular disease in overweight populaiton [36, 37]. Further studies are needed to clarify this issue.

The current study assessed the positive association between the urinary Na/K ratio and carotid atherosclerosis in a large relatively young Chinese sample. However, our study also has several limitations. First, this study did not use the gold standard 24-h urine test to evaluate sodium and potassium intake. Though this formula is the least biased among the INTERSALT and Tanaka methods [38–40], our estimates might not represent actual urinary sodium and potassium intake levels [41, 42]. Second, only one institution of physical examination in Changsha was include in current study. Hence, our results may not be generalizable to the rest of the Chinese population. Third, although the study adjusted for major sociodemographic characteristics and cardio metabolic factors, residual confounders, such as physical activity, and estimated glomerular filtration rate were not completely ruled out, which might mask or attenuate the true associations. Fourth, diabetes was diagnosed by medical history and fasting serum glucose, which leads to a low prevalence of diabetes. Last, due to the nature of a cross-sectional study, the current study was unable to determine any causal relationship between carotid artery atherosclerosis and the Na/K ratio. The exact causal relationship between the urinary excretion of Na/K ratio and carotid atherosclerosis needs further verification.

## Conclusions

Our study showed that high estimated excretion levels of urinary sodium and/or low estimated excretion levels of urinary potassium might be associated with an increased presence of carotid atherosclerosis in Chinese individuals.

## Supplementary Information

**Additional file 1: Table S1.** Characteristics of participants without carotid artery plaque by different Na/K ratios

**Additional file 2: Table S2** Adjusted associations of carotid IMT in different Na/K ratio subgroups*

## Abbreviations

eUNaE: Estimated urinary sodium excretion
eUKE: Estimated urinary potassium excretion
IMT: Intima-media thickness
Na/K ratio: The ratio of estimated urinary sodium vs. potassium excretion
BMI: Body mass index
CVD: Cardiovascular disease
SBP: Systolic blood pressure
DBP: Diastolic blood pressure
FSG: Fasting serum glucose
TC: Total cholesterol
TGs: Triglycerides
LDL-C: Low-density lipoprotein cholesterol
HDL-C: High-density lipoprotein cholesterol
CCA: Common carotid artery
BIF: Bifurcation carotid artery
SCr: Serum creatinine
IRB: Institutional Review Board
CIs: Confidence intervals
CHD: Chronic heart disease
