# Supplementary material for: The association of carotid artery atherosclerosis with the estimated excretion levels of urinary sodium and potassium and their ratio in Chinese adults
Source: Nutr J. 2021 Jun 6;20:50. doi: 10.1186/s12937-021-00710-8 (PMC8182948; doi:10.1186/s12937-021-00710-8)
Supplement: Supplementary file 1 — Additional file 1: Table S1. Characteristics of participants without carotid artery plaque by different Na/K ratios [file 12937_2021_710_MOESM1_ESM.doc]

**Table S1. Characteristics of participants** without carotid artery plaque by different Na/K ratios

| **Characteristics**  **(mean ± SD)** | **Total**  **(N=9,444)** | **Subgroup 1**  **Na/K ratio**  **0.0-1.0**  **(N=126)** | **Subgroup 2**  **Na/K ratio**  **1.1-2.0**  **(N=4,346)** | **Subgroup 3**  **Na/K ratio**  **2.1-3.0**  **(N=4,396)** | **Subgroup 4**  **Na/K ratio**  **3.1-4.0**  **(N=496)** | **Subgroup 5**  **Na/K ratio**  **>4.0**  **(N=80)** | ***P^1^*** | ***P_trend_*^2^** |
| --- | --- | --- | --- | --- | --- | --- | --- | --- |
| Age (years) | 45.79±9.71 | 46.15±8.85 | 45.76±9.60 | 45.82±9.74 | 45.30±10.55 | 48.61±10.13 | 0.082 | 0.67 |
| BMI (kg/m^2^)^7,8^ | 24.56±3.25 | 24.42±3.26 | 24.36±3.26 | 24.72±3.21 | 24.93±3.39 | 24.88±3.17 | <0.001 | <0.001 |
| SBP (mmHg)^4-11^ | 123.04±15.23 | 119.64±15.70 | 120.99±14.85 | 124.62±15.14 | 126.80±15.90 | 128.79±18.35 | <0.001 | <0.001 |
| DBP (mmHg)^4-11^ | 76.01±11.31 | 74.25±11.09 | 74.80±11.03 | 76.92±11.40 | 78.42±11.75 | 79.62±11.10 | <0.001 | <0.001 |
| FSG (mmol/L) | 5.56±1.21 | 5.61±1.39 | 5.57±1.31 | 5.54±1.11 | 5.56±1.05 | 5.62±1.21 | 0.77 | 0.47 |
| TC (mmol/L) | 5.05±0.97 | 5.00±1.16 | 5.05±0.97 | 5.05±0.96 | 5.05±0.98 | 5.01±0.85 | 0.98 | 0.99 |
| TG (mmol/L)^6,8,9,11,12^ | 1.90±1.85 | 1.76±1.28 | 1.85±1.72 | 1.92±1.89 | 2.09±2.41 | 2.59±2.87 | <0.001 | <0.001 |
| LDL-C (mmol/L)^9,11^ | 2.85±0.83 | 2.82±0.95 | 2.87±0.82 | 2.85±0.82 | 2.81±0.81 | 2.65±0.85 | 0.11 | 0.04 |
| HDL-C (mmol/L)^9^ | 1.35±0.31 | 1.36±0.34 | 1.35±0.32 | 1.34±0.31 | 1.33±0.29 | 1.28±0.28 | 0.06 | 0.01 |
| Estimated UNa (g/day) ^3-13^ | 4.32±1.15 | 2.06±0.60 | 3.70±0.88 | 4.83±0.94 | 5.68±1.02 | 5.35±1.47 | <0.001 | <0.001 |
| Estimated UK (g/day) ^4-13^ | 2.13±0.46 | 2.34±0.58 | 2.26±0.46 | 2.05±0.39 | 1.73±0.32 | 1.10±0.37 | <0.001 | <0.001 |
| Na/K ratio | 2.09±0.61 | 0.88±0.12 | 1.64±0.25 | 2.37±0.26 | 3.30±0.25 | 5.06±1.17 | <0.001 | <0.001 |
| CCA-IMT (mm)^3-6,9,11,12^ | 0.68±0.13 | 0.62±0.16 | 0.68±0.10 | 0.68±0.11 | 0.68±0.10 | 0.70±0.11 | <0.001 | 0.02 |
| BIF-IMT (mm) ^3-6,9,11,12^ | 0.83±0.18 | 0.76±0.22 | 0.83±0.18 | 0.83±0.18 | 0.83±0.18 | 0.91±0.17 | <0.001 | <0.001 |
| Male sex (%) ^4,7^ | 59.9 | 50.8 | 56.5 | 63.1 | 63.9 | 55.0 | <0.001 | <0.001 |
| Current alcohol users (%) | 32.4 | 24.2 | 31.4 | 33.6 | 33.9 | 32.5 | 0.04 | 0.01 |
| Current smokers (%) | 25.7 | 27.8 | 26.5 | 24.9 | 26.4 | 23.8 | 0.45 | 0.16 |
| Hypertension (%)^7-9,11,14^ | 26.1 | 25.4 | 23.3 | 27.9 | 31.0 | 43.8 | <0.001 | <0.001 |
| Dyslipidemia (%)^15^ | 34.9 | 37.3 | 35.0 | 35.0 | 32.1 | 41.3 | 0.47 | 0.62 |
| Diabetes mellitus (%)^16^ | 6.0 | 9.5 | 6.2 | 5.8 | 6.0 | 6.3 | 0.51 | 0.33 |
| CVD (%) | 1.0 | 0.0 | 1.1 | 0.8 | 1.0 | 1.3 | 0.53 | 0.58 |

Note: SD, standard deviation; CCA-IMT, common carotid artery intima-media thickness; BIF-IMT, bifurcation carotid artery intima-media thickness; BMI, body mass index; SBP, systolic blood pressure; DBP, diastolic blood pressure; FSG, fasting serum glucose; TC, total cholesterol; TG, triglycerides; LDL-C, low-density lipoprotein cholesterol; HDL-C, high-density lipoprotein cholesterol; UNa, urinary sodium excretion; UK, urinary potassium excretion; UCr, urinary creatinine excretion

^1^ *P* were obtained across five Na/K ratio subgroups among those without carotid plaque using by using analysis of variance (ANOVA) for continuous variables and the chi-square test for categorical variables.

^2^ *P_trend_* were obtained across five Na/K ratio subgroups among those without carotid plaque using generalized linear models for continuous variables and logistic regression for categorical variables.

^3^ Pairwise comparisons: subgroup 1 vs subgroup 2, *P*<0.05

^4^ Pairwise comparisons: subgroup 1 vs subgroup 3, *P*<0.05

^5^ Pairwise comparisons: subgroup 1 vs subgroup 4, *P*<0.05

^6^ Pairwise comparisons: subgroup 1 vs subgroup 5, *P*<0.05

^7^ Pairwise comparisons: subgroup 2 vs subgroup 3, *P*<0.05

^8^ Pairwise comparisons: subgroup 2 vs subgroup 4, *P*<0.05

^9^ Pairwise comparisons: subgroup 2 vs subgroup 5, *P*<0.05

^10^ Pairwise comparisons: subgroup 3 vs subgroup 4, *P*<0.05

^11^ Pairwise comparisons: subgroup 3 vs subgroup 5, *P*<0.05

^12^ Pairwise comparisons: subgroup 4 vs subgroup 5, *P*<0.05

^13^ 24-hour urinary sodium and potassium, and creatinine levels were estimated using the Kawasaki formula.

^14^ Hypertension was defined as self-reported hypertension diagnosed by a physician, self-reported regular use of antihypertensive medications, or systolic/diastolic blood pressure at recruitment ≥ 140/90 mmHg.

^15^ Dyslipidemia was defined as meeting any of the following criteria: 1) TC≥6.22 mmol/L; 2) LDL-C≥4.14 mmol/L; 3) HDL-C<1.04 mmol/L; 4) TG ≥2.26 mmol/L; 5) self-reported dyslipidemia or use of lipid-lowering medications;

^16^ Diabetes mellitus was defined as self-reported diabetes diagnosed by a physician, self-reported regular use of antidiabetic medications, or fasting glucose at recruitment ≥ 7.0 mmol/L.
