# Supplementary material for: The association of carotid artery atherosclerosis with the estimated excretion levels of urinary sodium and potassium and their ratio in Chinese adults
Source: Nutr J. 2021 Jun 6;20:50. doi: 10.1186/s12937-021-00710-8 (PMC8182948; doi:10.1186/s12937-021-00710-8)
Supplement: Supplementary file 2 — Additional file 2: Table S2 Adjusted associations of carotid IMT in different Na/K ratio subgroups* [file 12937_2021_710_MOESM2_ESM.docx]

Table S2 Adjusted associations of carotid IMT in different Na/K ratio subgroups*

| Variables | Na/K ratio | | |
| --- | --- | --- | --- |
| CCA-IMT | β | 95% CI | P |
| Na/K ratio 0.0-1.0（n=126） | 0.880 | 0.709, 1.050 | <0.01 |
| Na/K ratio 1.0-2.0（n=4,346） | 0.003 | -0.008, 0.014 | 0.58 |
| Na/K ratio 2.0-3.0（n=4,396） | 0.002 | -0.008, 0.012 | 0.65 |
| Na/K ratio 3.0-4.0（n=496） | -0.005 | -0.036, 0.026 | 0.74 |
| Na/K ratio >4.0（n=80） | 0.002 | -0.018, 0.022 | 0.85 |
| BIF-IMT | β | 95% CI | P |
| Na/K ratio 0.0-1.0（n=126） | 1.063 | 0.797, 1.329 | <0.01 |
| Na/K ratio 1.0-2.0（n=4,346） | 0.012 | -0.007, 0.031 | 0.21 |
| Na/K ratio 2.0-3.0（n=4,396） | 0.001 | -0.016,0.019 | 0.88 |
| Na/K ratio 3.0-4.0（n=496） | -0.009 | -0.064, 0.046 | 0.75 |
| Na/K ratio >4.0（n=80） | 0.006 | -0.025, 0.036 | 0.71 |

* adjusted for age, gender, body mass index, smoking, alcohol consumption, hypertension, diabetes mellitus, dyslipidemia and cardiovascular disease.
